# Remote Ischaemic Preconditioning Reduces Kidney Injury Biomarkers in Patients Undergoing Open Surgical Lower Limb Revascularisation: A Randomised Trial

**DOI:** 10.1155/2020/7098505

**Published:** 2020-01-23

**Authors:** Teele Kasepalu, Karl Kuusik, Urmas Lepner, Joel Starkopf, Mihkel Zilmer, Jaan Eha, Mare Vähi, Jaak Kals

**Affiliations:** ^1^Department of Surgery, Institute of Clinical Medicine, University of Tartu, Estonia; ^2^Department of Biochemistry, Institute of Biomedicine and Translational Medicine, Centre of Excellence for Genomics and Translational Medicine, University of Tartu, Estonia; ^3^Department of Cardiology, Institute of Clinical Medicine, University of Tartu, Estonia; ^4^Tartu University Hospital, Estonia; ^5^Department of Anaesthesiology and Intensive Care, Institute of Clinical Medicine, University of Tartu, Estonia; ^6^Department of Mathematics and Statistics, Faculty of Science and Technology, University of Tartu, Estonia

## Abstract

**Background and Aims:**

Perioperative kidney injury affects 12.7% of patients undergoing lower limb revascularisation surgery. Remote ischaemic preconditioning (RIPC) is a potentially protective procedure against organ damage and consists of short nonlethal episodes of ischaemia. The main objective of this substudy was to evaluate the effect of RIPC on kidney function, inflammation, and oxidative stress in patients undergoing open surgical lower limb revascularisation. *Materials and Methods*. This is a subgroup analysis of a randomised, sham-controlled, double-blinded, single-centre study. A RIPC or a sham procedure was performed noninvasively along with preparation for anaesthesia in patients undergoing open surgical lower limb revascularisation. The RIPC protocol consisted of 4 cycles of 5 minutes of ischaemia, with 5 minutes of reperfusion between every episode. Blood was collected for analysis preoperatively, 2, 8, and 24 hours after surgery, and urine was collected preoperatively and 24 hours after surgery.

**Results:**

Data of 56 patients were included in the analysis. Serum creatinine, cystatin C, and beta-2 microglobulin increased, and eGFR decreased across all time points significantly more in the sham group than in the RIPC group (*p* = 0.021, *p* = 0.021, *p* = 0.021, *p* = 0.021, *p* = 0.021,

**Conclusions:**

Our finding of reduced release of kidney injury biomarkers may indicate the renoprotective effect of RIPC in patients undergoing open surgical lower limb revascularisation. The trial is registered with ClinicalTrials.gov NCT02689414.

## 1. Introduction

Perioperative kidney injury has been found to affect 12.7% of patients undergoing lower limb revascularisation surgery, and only 23.8% of them have a prior history of chronic kidney disease [1]. In addition to chronic kidney failure, patients with peripheral artery disease (PAD) have frequently multiple risk factors for acute kidney injury (AKI) such as smoking, other cardiovascular diseases, diabetes mellitus, and usage of nephrotoxic medication, including painkillers [2, 3]. In addition, systemic stress response due to surgery induces haemodynamic changes including changes in renal blood flow, which promotes kidney injury. Additionally, during lower limb revascularisation, there occurs ischaemia-reperfusion damage and nephrotoxic myoglobin is released to the circulation. AKI is associated with worse outcome and especially with higher risk for cardiovascular disease [1, 4]. Even small changes in creatinine are associated with higher mortality, morbidity, and a greater economic burden [5].

Some precautions and recommendations help to reduce kidney injury, e.g., avoiding nephrotoxic agents, hyperglycemia, and hypovolemia and close haemodynamic monitoring. Yet perioperative renoprotective agents and interventions are still lacking. Remote ischaemic preconditioning (RIPC), which consists of short nonlethal episodes, has been found to reduce organ damage including kidney injury. The main organoprotective effect of RIPC is considered to arise from attenuating the damage due to ischaemia-reperfusion. Ischaemia-reperfusion injury is believed to be mediated by calcium overload, oxidative stress (OxS), and inflammation [6].

Although early studies have shown a renoprotective effect of RIPC in patients undergoing vascular surgery [7, 8], the last 3 large studies report no benefit [9–11]. Still, recent meta-analyses have indicated a renoprotective effect of RIPC in patients undergoing cardiac surgery [12, 13].

This is a substudy within our large clinical trial conducted for evaluating the effect of RIPC on vascular stiffness and end-organ damage [14, 15]. Among our secondary aims was to investigate changes in the levels of traditional and novel kidney function biomarkers and markers of inflammation and OxS. We hypothesised that RIPC may reduce the leakage of kidney function biomarkers and the markers of inflammation and OxS in patients undergoing open surgical lower limb revascularisation, and the aim of the current study was to test this hypothesis.

## 2. Methods

### 2.1. Study Groups and Eligibility

The study was carried out at the Department of Vascular Surgery, Clinic of Surgery, at Tartu University Hospital from January 1, 2016, to February 8, 2018. This clinical trial was randomised, double-blinded, and sham-controlled. Patients who underwent elective open surgical lower limb revascularisation (aortofemoral or/and femoropopliteal segments) for claudication or critical limb ischaemia and who gave their full informed consent for participation were enrolled in the current study. The study was approved by the Research Ethics Committee of the University of Tartu and was registered in the ClinicalTrials.gov database (NCT02689414).

The exclusion criteria were the following: age under 18 years, pregnancy, known malignancy in the past 5 years, permanent atrial fibrillation or flutter, symptomatic upper limb atherosclerosis, need for oxygen therapy at home, estimated preoperative glomerular filtration rate (eGFR) < 30 mL/min/1.73 m^2^, myocardial infarction in the past month, previous history of upper limb vein thrombosis or vascular surgery in the axillary region, and inability to follow the study regimen.

### 2.2. Randomisation

Patients were randomly and equally assigned to the sham or RIPC group in parallel. A stratified block design with block size 2 or 4 was used. A random allocation sequence was generated by the computer program WINPEPI (PEPI-for-Windows). Patients were stratified according to age (under or over 65 years) and the American Society of Anesthesiologists' (ASA) physical status classes 2, 3, or 4. Randomisation and opaque sealed envelopes were prepared by a third party. The envelopes were opened immediately before intervention.

### 2.3. Intervention

The protocol of RIPC consisted of four 5-minute episodes of upper limb ischaemia. For achieving ischaemia, pressure in the blood pressure cuff was raised for 5 minutes to 200 mmHg or, when the patient's blood pressure exceeded 180 mmHg, to a value that was 20 mmHg higher than the value of systolic blood pressure. In the sham group, pressure in the cuff was equal to venous pressure (10–20 mmHg). Between all episodes, there was a 5-minute period of reperfusion. Intervention was started along with preparation. Participation in the study did not influence any other aspects of surgery, anaesthesia, or medication use.

### 2.4. Blinding

The patient, patient's physician, surgeon, anaesthesiologist, and everyone else in the surgical team were blinded to the study intervention. The manometer scale was kept shielded.

### 2.5. Outcomes

Blood samples were collected in the morning of surgery and at 2 hours, 8 hours, and approximately 24 hours of surgery, and urine samples were collected in the morning of surgery and 24 hours after surgery. The last blood and urine collection was set as close as possible to 24 hours after surgery on condition that the patient had fasted for at least 3 hours. The level of creatinine, urea, cystatin C, beta-2 microglobulin, and neutrophil gelatinase-associated lipocalin (NGAL) in blood was measured at all of the abovementioned time points, and the level of interleukin 18 (IL-18) and oxidized low-density lipoprotein (oxLDL) was measured preoperatively and at 8 hours and approximately 24 hours of surgery. Estimated GFR (eGFR) was calculated using the Chronic Kidney Disease Epidemiology Collaboration (CKD-EPI) equation. Urine samples were analysed for the level of kidney injury molecule 1 (KIM-1), liver-type fatty acid binding protein (L-FABP), isoprostanes, and creatinine. The level of isoprostanes was corrected for spot urine creatinine.

AKI was diagnosed based on a more than 26.5 *μ*mol/L increase in creatinine within the trial, according to the Kidney Disease Improving Global Outcomes (KDIGO) criteria.

All patients were questioned about their previous and current health issues and medication use. In addition, an electronic health database and surgery protocols were used for completing the anamnesis.

### 2.6. Statistical Analysis

Continuous variables were compared using Student's *t*-test or the Wilcoxon rank-sum test as appropriate. For statistical analysis of multiple repeated measures, analysis of variance (ANOVA) or multivariate analysis of variance (MANOVA) was used. Categorical variables were compared with the chi-squared test.

*p* values under 0.05 were considered significant. As the primary outcome of the study was arterial stiffness parameters, calculation of sample size was based on AIx [15]. For both groups, the calculated sample size was 44.

## 3. Results

**Figure 1:**
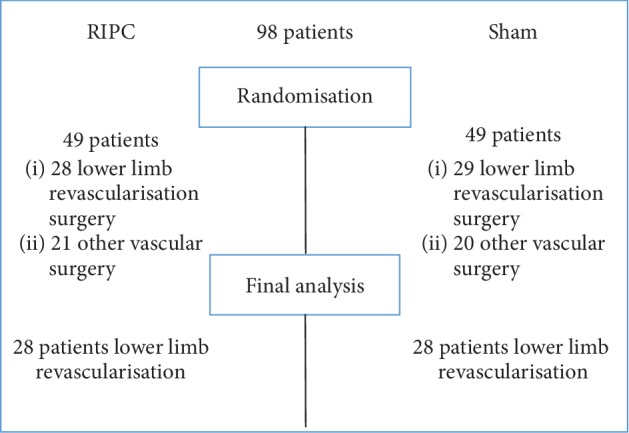
Patients' flow chart. After randomisation, there were 28 patients in the RIPC group and 29 patients in the sham group who underwent lower limb revascularisation surgery; of these patients, 1 patient dropped out from the sham group.

Fifty-seven patients undergoing open lower limb revascularisation surgery were enrolled in the trial and randomised into study groups. After dropout, 28 patients were included both in the RIPC group and the sham group. A detailed patient flow is shown in Figure 1. In 3 patients from the RIPC group and in 2 patients from the sham group, blood collection was missed either at 2 hours or 8 hours of surgery. In 3 patients from the sham group, urine was not collected 24 hours after surgery. In the sham group, there were more women (3 vs. 10 patients, *p* value 0.058) and diabetic patients (2 vs. 6, *p* = 0.252) and more patients were administered propofol (7 vs. 12, *p* = 0.259), but there were no statistically significant differences between the groups (*p* > 0.05, Table 1). No patient experienced side effects of RIPC or found the procedure intolerable.

The mean time from the end of intervention to the beginning of surgery was 40 minutes in the RIPC group and 33 minutes in the sham group (*p* = 0.257). No adverse events due to RIPC were reported. In 4 patients (14%) from the sham group and in 1 (4%) patient from the RIPC group, AKI could be diagnosed based on the KDIGO criteria (*p* = 0.604).

### 3.1. Serum Kidney Function Markers

There was a significant change in an overall increase in creatinine (*p* = 0.011), eGFR (*p* = 0.007), cystatin C (*p* = 0.038), and beta-2 microglobulin (*p* = 0.013) in the sham group through all time points (Figure 1). There was a significant change in an overall decrease in urea (*p* = 0.050, Figure 1) and a significant increase in NGAL (*p* = 0.001) in the RIPC group through all time points. The change in creatinine, urea, cystatin C, and beta-2 microglobulin was not statistically significant in the RIPC group (*p* > 0.05), and the change in urea and NGAL was not statistically significant in the sham group (*p* > 0.05) (Table 2).

**Figure 2:**
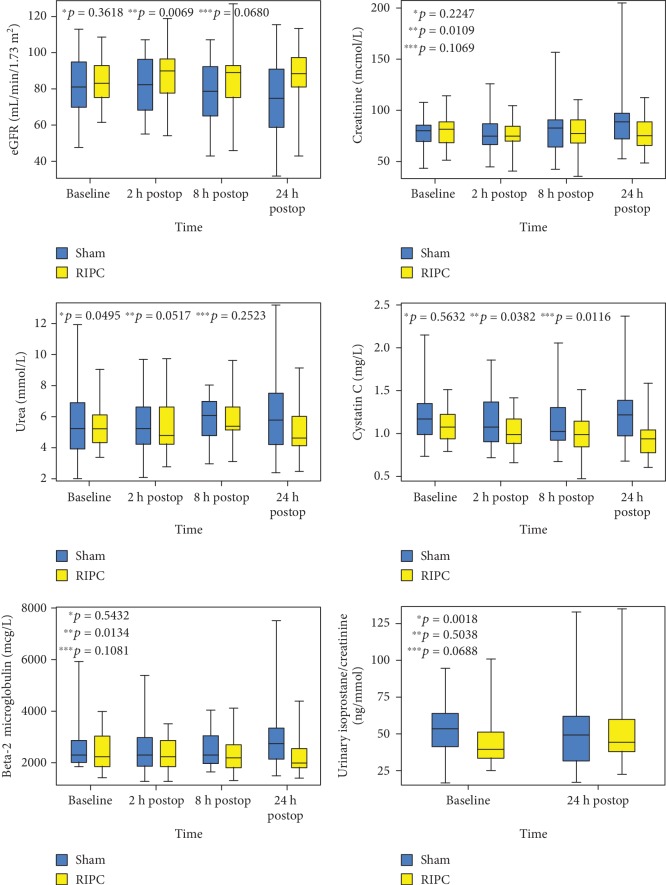
Statistically significant changes between the sham group and the RIPC group—changes in creatinine, estimated glomerular filtration rate (eGFR), urea, cystatin C, beta-2 microglobulin, and the ratio of urinary isoprostanes to creatinine. ^∗^*p* value for the change in the RIPC group; ^∗∗^*p* value for the change in the sham group; ^∗∗∗^*p* value for the changes between the groups.

Creatinine increased significantly more in the sham group than in the RIPC group (*p* = 0.021) through all time points (from baseline to 2, 8, and 24 hours after surgery). The eGFR decreased significantly more in the sham group (*p* = 0.015). There was no significant change in urea between the groups through all time points (*p* = 0.067). The change in cystatin C and beta-2 microglobulin was significantly different between the groups (*p* values 0.021 and 0.024, respectively). Creatinine, cystatin C, and beta-2 microglobulin increased and eGFR decreased significantly more in the sham group than in the RIPC group through all time points (*p* = 0.021, *p* = 0.024, *p* = 0.021, and *p* = 0.015, respectively). Changes in creatinine, eGFR, urea, cystatin C, and beta-2 microglobulin through all time points are presented in Figure 2. The change in NGAL levels between the groups was nonsignificant (*p* value 0.174).

When comparing the two time points, baseline and 24 hours after surgery, the change in creatinine, eGFR, urea, cystatin C, and beta-2 microglobulin was significantly different between the groups (*p* < 0.05, Table 2).

### 3.2. Urinary Kidney Injury Markers

KIM-1 increased statistically significantly both in the RIPC group (change median 930 pg/mL, IQR -81−3078, *p* = 0.001) and in the sham group (change median 1238 pg/mL, IQR -124−3097, *p* = 0.006), but there was no difference between the groups (*p* = 0.935). The change in L-FABP levels was not statistically significant in either group, nor was there any difference between the groups (*p* = 0.710, Table 2).

### 3.3. Oxidative Stress and Inflammation Markers

The change in the level of urinary isoprostanes, corrected for creatinine, was statistically different between the groups (*p* = 0.039, Figure 2); in the RIPC group, median change was 5.4, IQR -1−18, and in the sham group, median change was 0.3, IQR -13−17. The changes in the levels of hs-CRP, IL-18, adiponectin, and oxLDL were statistically significant both in the RIPC group (*p* values < 0.001) and in the sham group (*p* values < 0.001), but their magnitude in both groups was similar (*p* values 0.504, 0.649, and 0.534, respectively). The change in oxLDL did not differ among the groups either (*p* = 0.880) and was nonsignificant both in the RIPC group (*p* = 0.220) and in the sham group (*p* = 0.216). The changes from the baseline to 24 hours after surgery are presented in Table 2.

## 4. Discussion

The results of the study showed a renoprotective effect of RIPC in patients undergoing open surgical lower limb revascularisation. The increase in creatinine, urea, cystatin C, and beta-2 microglobulin in serum was statistically lower in the RIPC group compared to the sham group. There was also a statistically significant increase in the urinary isoprostane/creatinine ratio in the RIPC group compared to the sham group. However, there occurred no significant difference in the changes in serum NGAL, adiponectin, IL-18, oxLDL, and MPO nor urinary KIM-1 and isoprostanes between the RIPC group and the sham group.

Although perioperative kidney injury is related to higher mortality, cardiovascular morbidity, risk for chronic kidney injury, and longer need for hospitalisation, it has still remained an underdiagnosed and undertreated problem [16]. Multiple agents, such as N-acetylcysteine [17], levosimendan [18], and statins [19], have been studied regarding their renoprotective effect in the perioperative period, but without significant supportive evidence. In addition to RIPC, anaesthetic dexmedetomidine is also a promising renoprotective agent [20]. However, as most lower limb revascularisation operations are not performed under general anaesthesia, RIPC would be a clinically better option. Still, the results of previous studies about the effect of RIPC on renoprotection are contradictory.

Postoperative AKI has been found to occur in 12.7−49% of patients undergoing vascular surgery [1, 5]. Patients with coexisting PAD are at greater risk for AKI. In patients undergoing revascularisation surgery, ischaemia-reperfusion injury and rhabdomyolysis are inevitable, increasing the risk for kidney injury. However, in the RIPC group, there was a significant decrease of creatinine, urea, cystatin C, and beta-2 microglobulin median levels 24 hours after surgery, compared to the sham group, where these biomarkers even increased. Presumably, this decrease in the biomarkers is mainly associated with the protective effect of RIPC rather than dilution due to infusion therapy, as the infusion volume was similar during surgery in both RIPC and sham groups. In our study, multiple kidney function markers were measured at 3 time points during 24 hours after surgery. Following AKI, kidney function markers are expected to increase within 7 days. Based on the KDIGO criteria, an AKI is diagnosed on the basis of creatinine and urine output changes within 7 days. As our trial lasted only 24 hours after surgery, it was not possible to accurately evaluate the presence of AKI. According to our findings, AKI was present in at least 4 patients (14%) in the sham group and in 1 patient (4%) in the RIPC group. However, it is very likely that more patients developed AKI. Moreover, we excluded patients with eGFR < 30 mL/min/1.73m^2^, who were at greater risk for AKI. We found no difference in the increase of urinary L-FABP and KIM-1 between the groups. Yet in both groups, the median level of KIM-1 exceeded the normal range value of 59-2146 pg/mL [21] attaining a value above the reference. It has been found that even a rise of 1000 pg/mL in KIM-1 is associated with greater than 12-fold risk for acute tubular necrosis [22]. This indicates that kidney injury may affect over 50% of patients undergoing lower limb revascularisation surgery. KIM-1, NGAL, and L-FABP are considered to be more sensitive than traditional kidney function markers in detecting true kidney injury and not the capacity of filtration. Among the above three kidney injury markers, we found no differences between the groups. On the contrary, in the RIPC group, there was a significantly smaller increase in the markers describing glomerular filtration, such as urea, creatinine, and cystatin C. These differences can be explained by the fact that RIPC offers more protection to glomeruli than to tubules; or that changes in the level of KIM-1, NGAL, and L-FABP need not be simultaneously accompanied by changes in the markers of glomerular filtration, which is why statistical difference could have been missed.

Surgery induces a systemic inflammatory response, whose extent has considerable variability among individuals. More pronounced inflammatory response is associated with higher risk for AKI [23]. In our study, IL-18, adiponectin, hs-CRP, and oxLDL changed statistically significantly in both groups, but there was no difference between the groups. The only OxS marker that changed differently between the groups was the ratio of urinary isoprostanes to creatinine. It is recommended to correct isoprostane levels for urinary creatinine to avoid differences due to urine dilution [24]. Unexpectedly, isoprostanes, corrected for creatinine, increased in the RIPC group and decreased in the sham group. This finding suggests that postischaemic vasodilatation may have induced a greater synthesis of isoprostanes from prostaglandines in the RIPC group. At the same time, the level of isoprostanes/creatinine was at the baseline and remained in the normal range, according to endemic reference values, after surgery. Previous studies have concluded that RIPC reduces OxS [25, 26]. There is evidence that RIPC prevents ischaemia-reperfusion-induced renal dysfunction by decreasing MPO levels in rats [27]. Yet to our knowledge, there are no studies that have evaluated the effect of RIPC on the OxS markers adiponectin, IL-18, and oxLDL. The similar decrease in oxLDL in both groups can be explained with increased macrophage activity, owing to inflammatory response, as macrophages take up oxLDL. Also, the decrease in adiponectin level can be associated with inflammatory response. Adiponectin has been found to decrease under stress conditions, such as myocardial infarction [28], which supports our finding. There occurred an increase in the level of MPO, an early biomarker of inflammation [29], in both groups. All these changes indicate that lower limb revascularisation surgery induces high-grade OxS. However, the decrease in IL-18 level remains unclear. Despite the increase in the ratio of urinary isoprostanes to creatinine and the absence of the effect of RIPC on the other OxS markers, no definite conclusions could be drawn about the effect of RIPC on OxS because of the small size of the study sample. Considering the above facts, we cannot conclude that the effect of RIPC is mediated through MPO, oxLDL, IL-18, or adiponectin.

Even though surgery as well as surgery-related stress response can induce kidney injury, stress response *per se* can influence many, if not all, kidney function markers. Creatinine also increases along with rhabdomyolysis and urea increases along with stress response. In addition to being a kidney injury marker, NGAL is an acute phase protein released by neutrophils and increased in both groups similarly. Presumably, the level of NGAL is influenced by the inflammatory response to surgery rather than by kidney injury. The most reliable kidney injury marker in our study was evidently cystatin C, which is also influenced by systemic inflammatory response.

There are increasingly more studies that have concluded that propofol may abolish RIPC's effect [30, 31]. It has been found that patients who have not received propofol during anaesthesia were less likely to develop AKI [32]. As 25% in the RIPC group propofol was administered, the true effect of RIPC might be underestimated in our study.

Taken all together, we describe the detectable effect of RIPC in lowering kidney injury biomarkers. Based on this, RIPC might be a clinically valuable option to reduce the incidence of AKI especially because well-established procedures to decrease perioperative AKI are lacking. Reducing perioperative AKI could shorten hospital stay and ameliorate prognosis. Furthermore, RIPC is low-cost and based on our findings easily applicable and well tolerated by patients, which all ease the usage in clinical practice.

The main limitation of our study is its small sample size. Also, there were differences between the two groups, although they were statistically nonsignificant. The number of women was larger in the sham group, and the female gender is associated with higher risk for postoperative AKI [33]. Also, there were more diabetic patients in the sham group, and diabetes is known to increase the risk for AKI. These are major limitations as patients in the sham group had greater risk for AKI in many ways. However, as the effect of RIPC has been found to be diminished in diabetic patients, we might have been able to observe greater effect of RIPC. Moreover, some patients were administered propofol, which has been found to have negative impact to RIPC effect.

Taken together, our study supports the evidence about the renoprotective effect of RIPC. However, the heterogeneity and small size of the study population reduce its credibility. Larger studies are needed to clarify the true effect of RIPC in kidney protection in patients undergoing open surgical lower limb revascularisation.

## Figures and Tables

**Table 1:** Baseline and surgery characteristics.

	RIPC (*n* = 28)	Sham (*n* = 28)	*p* value
Age, years (SD)	67 (10)	66 (10)	0.625
Female, *n* (%)	3 (11%)	10 (36%)	0.058
Current smoker, *n* (%)	19 (68%)	15 (54%)	0.412
Diabetes, *n* (%)	2 (7%)	6 (21%)	0.252
ACEI or ARB, *n* (%)	10	16	0.180
Calcium channel blockers, *n* (%)	6	9	0.546
Contrast dye ≤ 7 days before surgery or ≤24 h after surgery, *n* (%)	17 (61%)	17 (61%)	1
Infusion during surgery, median (IQR)	1100 (1000−1525)	1150 (1000−1500)	0.622
Administration of propofol during surgery, *n* (%)	7 (25%)	12 (43%)	0.259
Fontaine class 2	10 (36%)	10 (36%)	1
Fontaine class 3	8 (29%)	9 (32%)	1
Fontaine class 4	8 (29%)	6 (21%)	0.546
Unclassifiable by Fontaine classification	1 (4%)	3 (11%)	0.604
Femoropopliteal bypass	14 (50%)	14 (50%)	1
Femoral artery endarterectomy	4 (14%)	4 (14%)	1
Aortobifemoral bypass	6 (21%)	5 (18%)	1
Other peripheral vascular surgery (iliofemoral and femorofemoral bypass)	0 (0%)	2 (7%)	0.472
Redo vascular surgery	4 (14%)	3 (11%)	1

*n*: number of patients; SD: standard deviation; ACEI: angiotensin-converting enzyme inhibitor; ARB: angiotensin II receptor blocker; Fontaine class: peripheral artery disease class according to Fontaine classification; IQR: interquartile range.

**Table 2:** Changes in kidney and oxidative stress biomarkers from baseline to 24 hours after surgery in the RIPC and sham groups.

	RIPC (*n* = 28)			Sham (*n* = 28)			RIPC vs. Sham *p* value
	Baseline	24 h after surgery	*p* value	Baseline	24 h after surgery	*p* value	
Serum creatinine (*μ*mol/L)	80 (68−88)	74 (64−88)	0.030	79 (69−85)	88 (71-95)	0.024	**0.003**
eGFR (mL/min/1.73m^2^)	84 (75−93)	89 (81−98)	0.037	82 (70-95)	75 (59−92)	0.016	**0.005**
Serum urea (mmol/L)	5.2 (4.4−6.2)	4.7 (4.1−6.1)	0.222	5.3 (4.0−6.9)	5.8 (4.2−7.6)	0.075	**0.041**
Serum cystatin C (mg/L)	1.1 (0.9−1.2)	0.9 (0.8−1.0)	0.0002	1.2 (1.0−1.4)	1.2 (1.0−1.4)	0.690	**0.022**
Serum beta-2 microglobulin (*μ*g/L)	2195 (1820−3005)	2000 (1775−2520)	0.184	2215 (2010−2850)	2720 (2100−3325)	0.062	**0.033**
Serum NGAL (ng/mL)	79 (68−94)	100 (82−118)	<0.0001	82 (71−106)	106 (80−166)	<0.0001	0.407
Urinary KIM-1 (pg/mL)	1343 (871-2046)	2689 (1324-4793)	0.001	1428 (776-2352)	3025 (1185-4629)	0.006	0.935
Urinary L-FABP (ng/mL)	1.2 (0.9-1.8)	1.2 (0.9-1.4)	0.640	1.0 (0.9-1.4)	1.0 (0.9-1.6)	0.814	0.710
Serum adiponectin (ng/mL)	5712 (3037−9063)	5179 (2651−7214)	<0.0001	5129 (2708−7244)	4697 (2604−6431)	<0.0001	0.744
Serum IL-18 (pg/mL)	294 (229−388)	277 (236−365)	<0.0001	292 (236−371)	265 (230−359)	<0.0001	0.794
Serum oxLDL (U/L)	67 (55−84)	52 (44−67)	<0.0001	60 (49−74)	47 (40−59)	<0.0001	0.942
Serum hs-CRP	3.5 (1.4-13.5)	43.9 (18.5-77.9)	<0.0001	3.9 (2.5-8.0)	62.1 (29.8-80.9)	<0.0001	0.209
Urinary isoprostanes/creatinine (ng/mmol)	40 (34-52)	45 (39-60)	0.002	54 (43-64)	51 (33-63)	0.504	**0.039**

eGFR: estimated glomerular filtration rate; NGAL: neutrophil gelatinase-associated lipocalin; oxLDL: oxidized low-density lipoprotein; hs-CRP: high-sensitivity C-reactive protein; KIM-1: kidney injury molecule 1; L-FABP: liver-type fatty acid binding protein.

## Data Availability

The data used to support the findings of this study are available from the corresponding author upon request.
